# Supplementary material for: Peptide collision cross sections of 22 post-translational modifications
Source: Anal Bioanal Chem. 2023 Sep 28;415(27):6633–45. doi: 10.1007/s00216-023-04957-4 (PMC10598134; doi:10.1007/s00216-023-04957-4)
Supplement: Supplementary file 1 — Supplementary file1 (DOCX 1995 KB) [file 216_2023_4957_MOESM1_ESM.docx]

# Supplementary Information

**Peptide collision cross sections of 22 post-translational modifications**

Andreas Will^1^, Denys Oliinyk^1^, Christian Bleiholder^2^, Florian Meier^1^


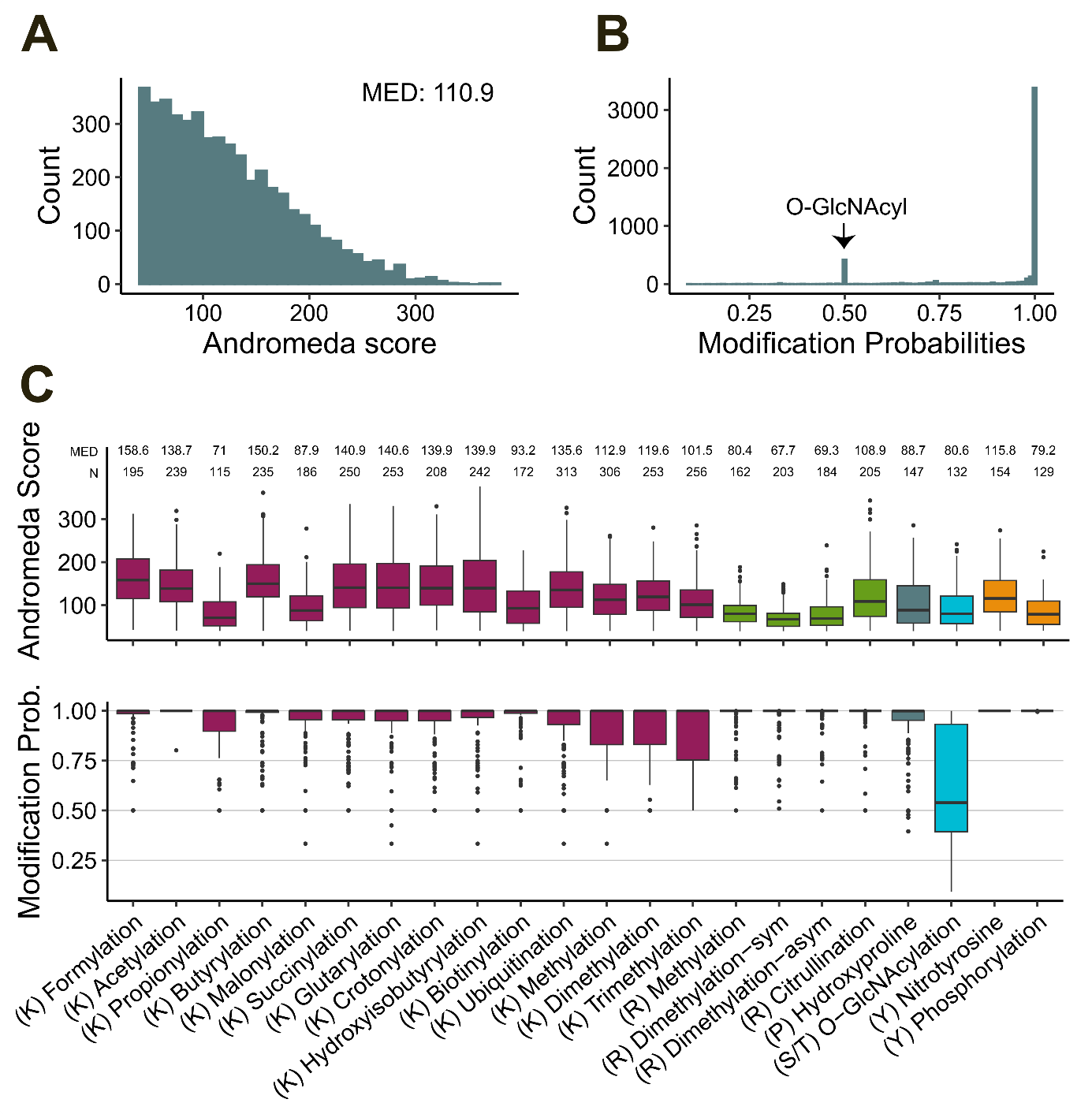


**Supplementary Figure 1. (A)** Andromeda score distribution of unique (highest intensity) combinations of sequence, charge state and modification (n = 4539, bin width = 10). **(B)** Same as A, but for modification localization probability (bin width = 0.01). **(C)** Modification specific boxplot of Andromeda score distributions (top) and modification localization probability (bottom). Boxplot elements: Interquartile range within boxes; median indicated by horizontal line; whiskers spanning 1.5 x interquartile range.


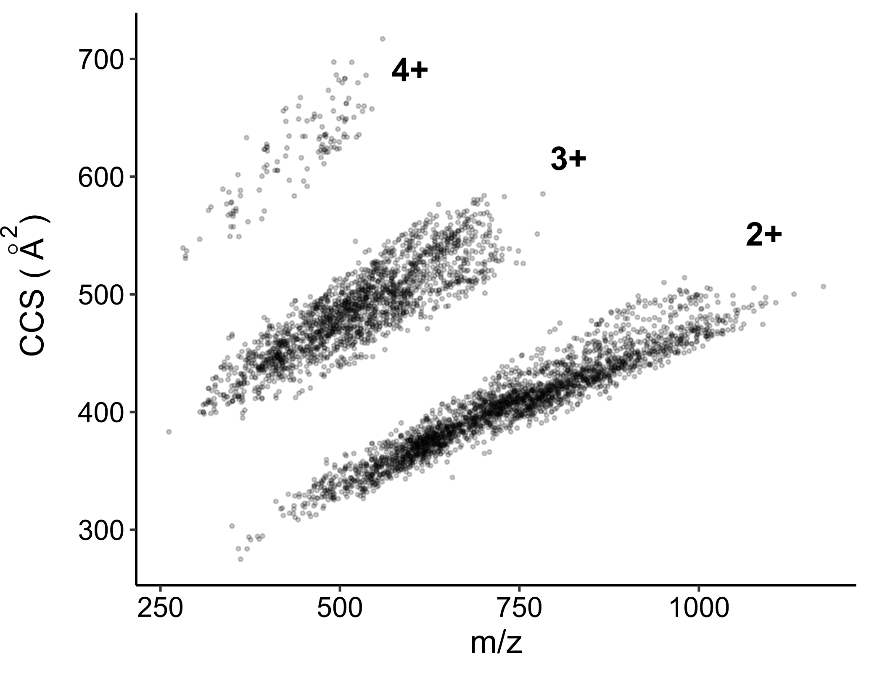


**Supplementary Figure 2.** Distribution of unique combinations of sequence, charge state and modification in the *m/z* vs. CCS space (n = 4,539).

**
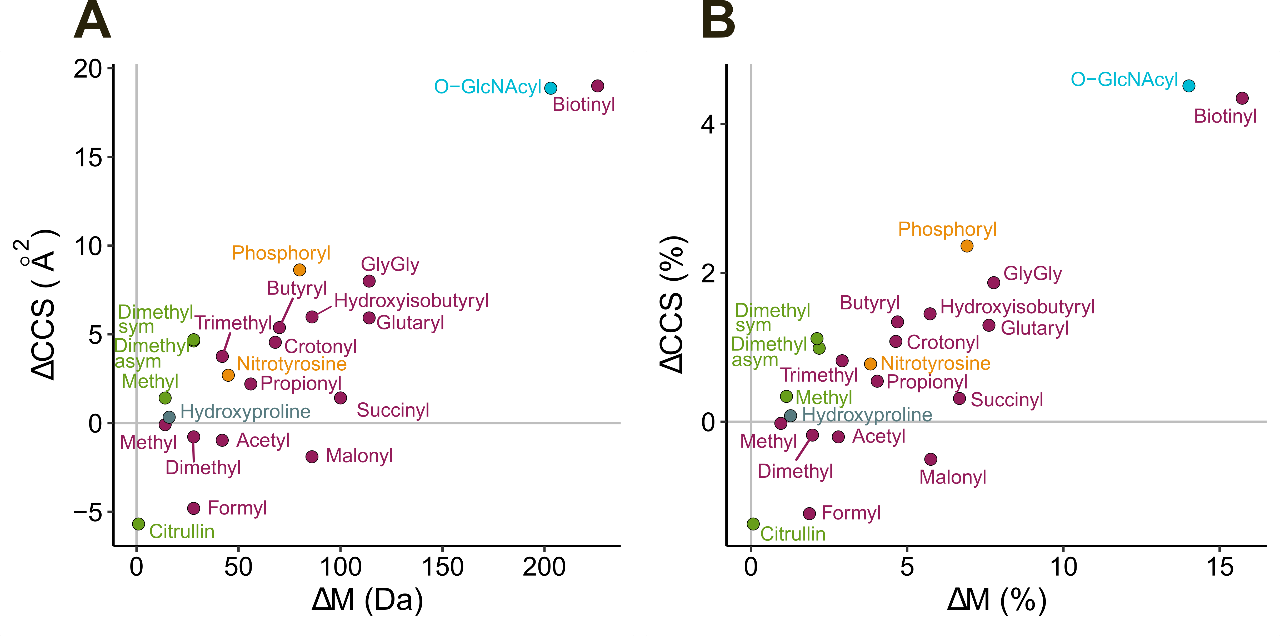
**

**Supplementary Figure 3**. Pairwise comparison of modified and unmodified peptide collision cross section (CCS). **(A)** Median ΔCCS values of all investigated modifications as a function of the molecular weight change (ΔM) **(C)** Same as B, but with relative values.


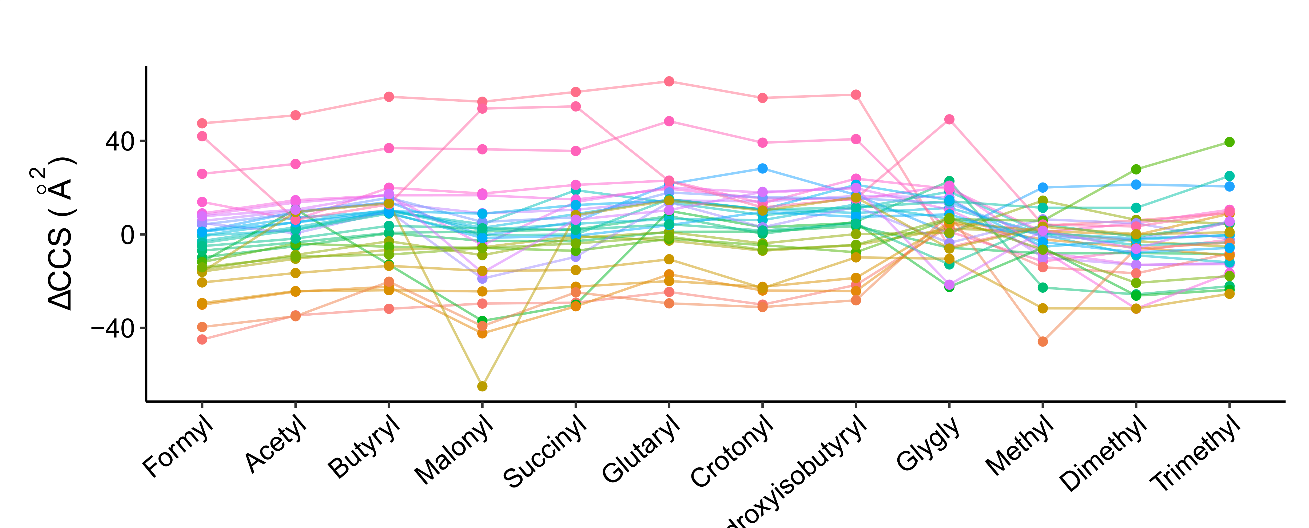


**Supplementary Figure 4.** ΔCCS values of individual peptide sequences for acyl type modifications on lysine and charge 3 (n = 45). Malonylation and biotinylation were excluded due to their low overlap.


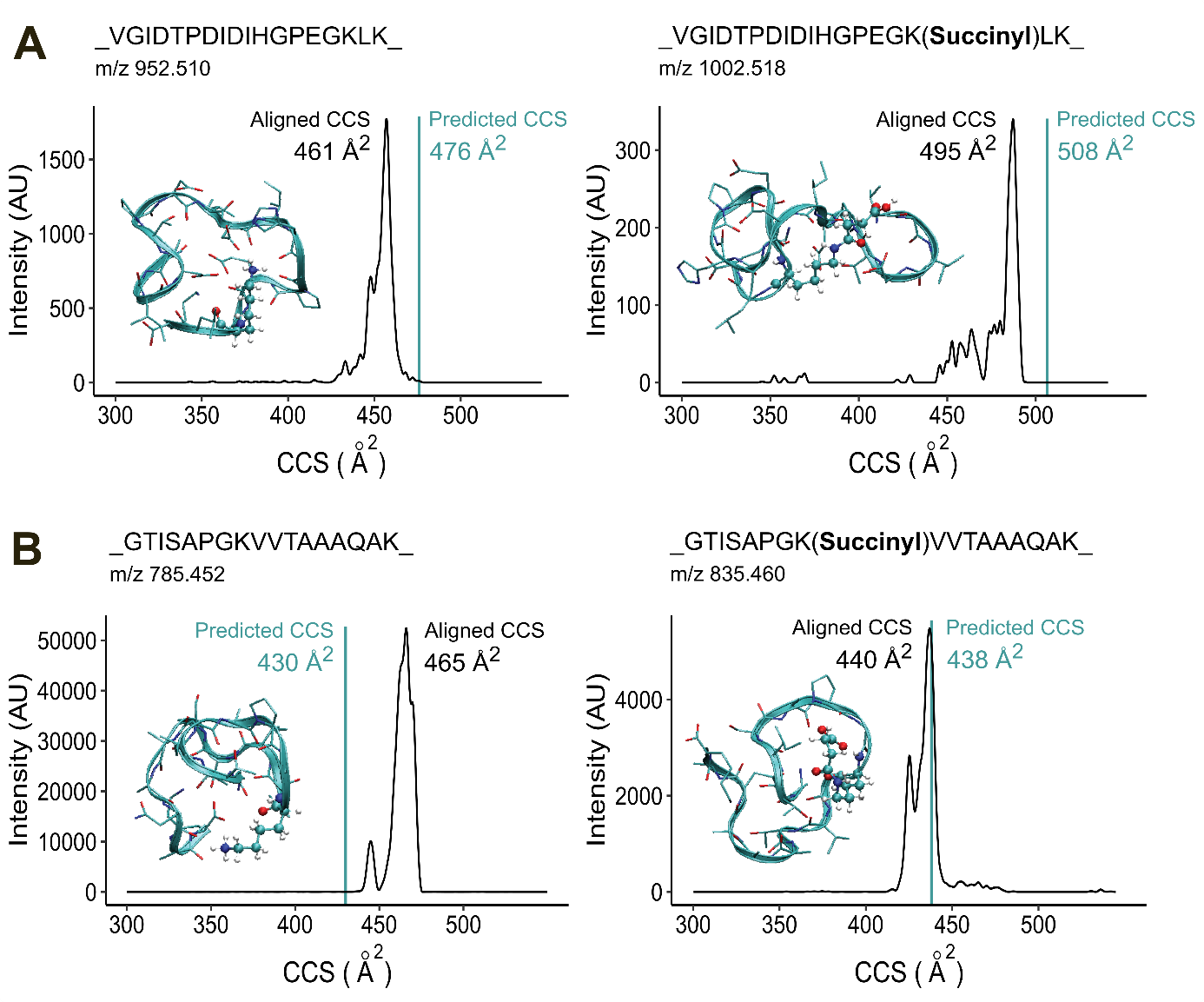


**Supplementary Figure 5.** **Experimental and modeled collision cross section (CCS) of two selected doubly-charged peptides with and without lysine succinylation.** Summed ion mobility spectra were extracted from a mass window of ± 0.005 Da and a retention time length of 0.1 min around the chromatographic peak apex. CCS axes were aligned as described in Methods and the annotated experimental CCS values were taken from the MaxQuant output for the most abundant ‘evidence’. Modeled peptide CCS values are indicated in the mobility spectra along with their predicted structures as obtained through molecular dynamics.


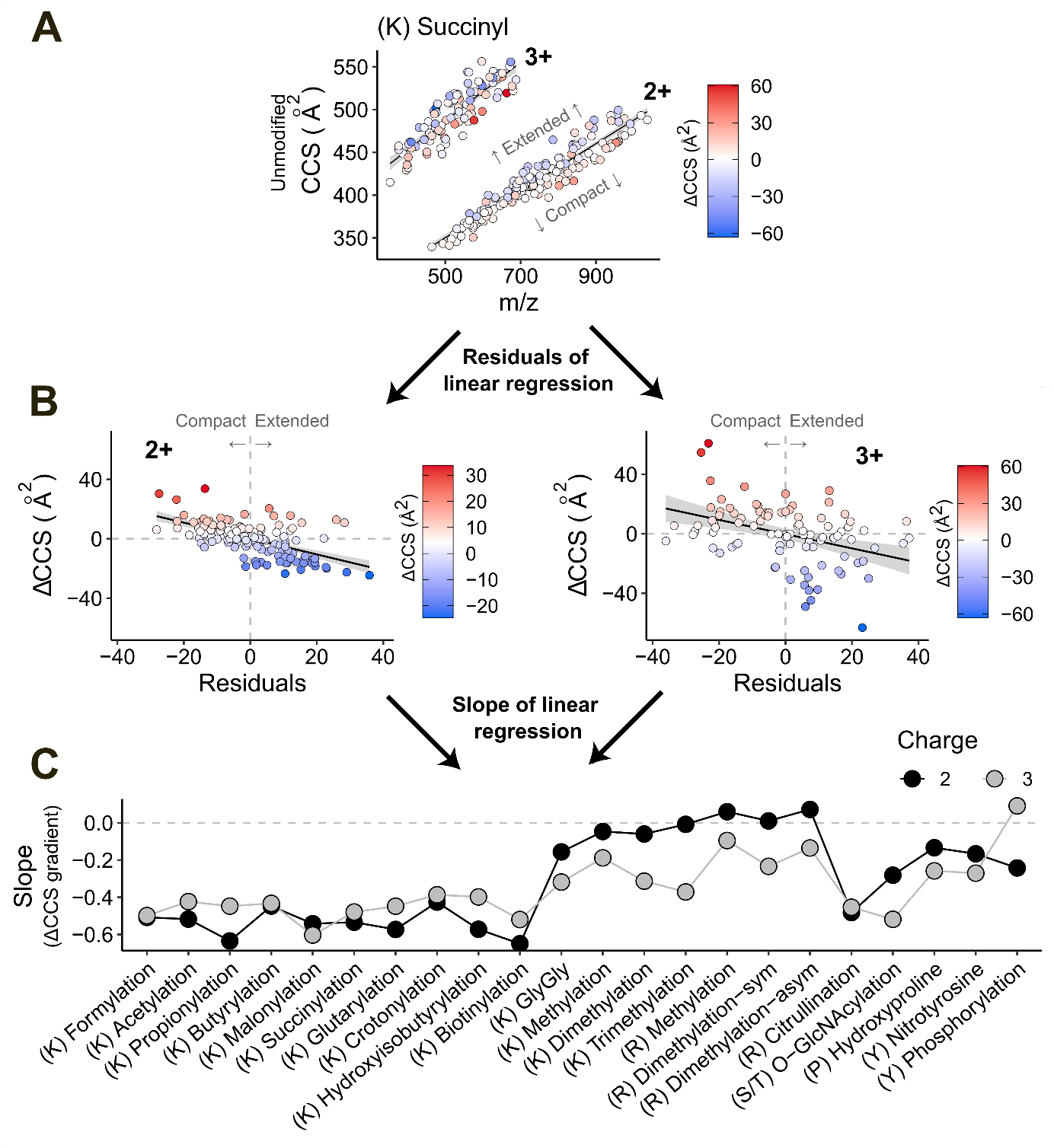


**Supplementary Figure 6. Distribution of modification-induced ΔCCS values within ion clouds.** (A) Peptide *m/z* vs. CCS distribution of unmodified peptides overlaid with the CCS shift induced by succinylation. Linear trend lines are fitted to both charge states. (B) ΔCCS vs. residuals of linear regression (taken from CCS vs. *m/z*, see panel A). A linear trend line is fitted to the graph. Its slope represents the ΔCCS gradient within the ion clouds shown in panel A. (C) Overview of ΔCCS gradients, as described in panel B, through all investigated PTMs.


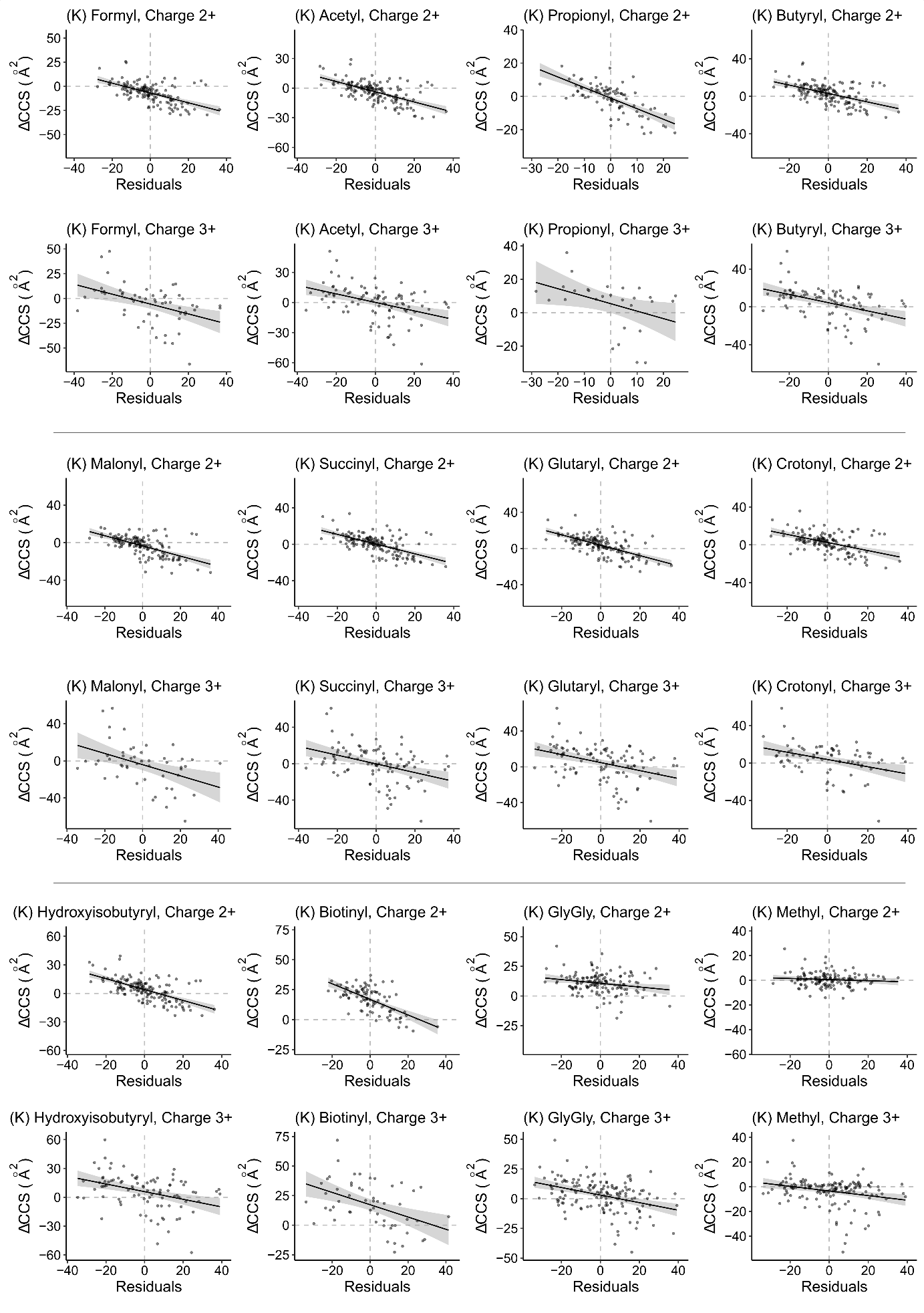


**Supplementary Figure 7.** ΔCCS vs. residuals of linear regression for all investigated modifications, separated by Charge 2 and 3 (see Suppl. Fig. 6).


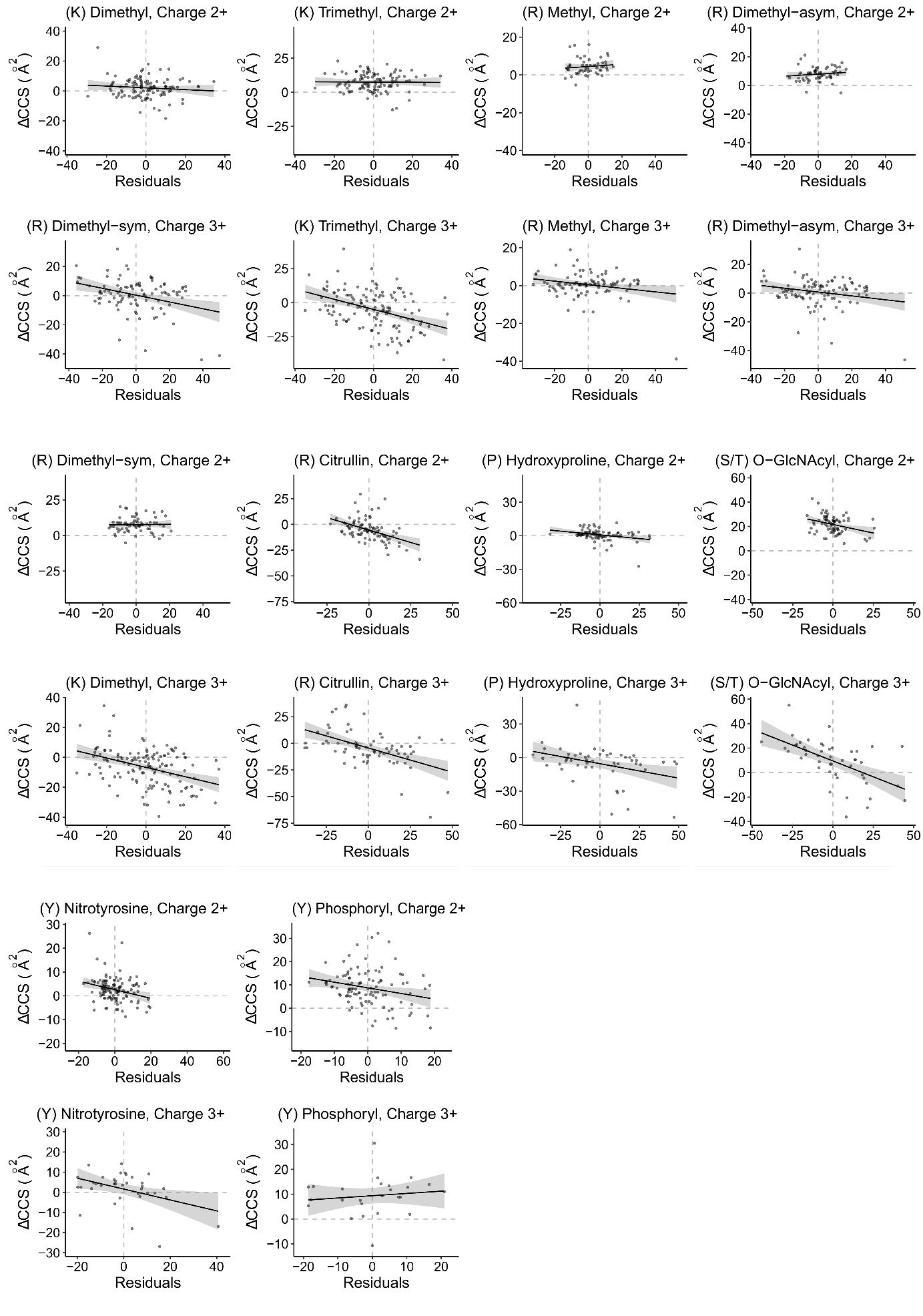


**Supplementary Figure 7 (continued).** ΔCCS vs. residuals of linear regression for all investigated modifications, separated by Charge 2 and 3 (see Suppl. Fig. 6).


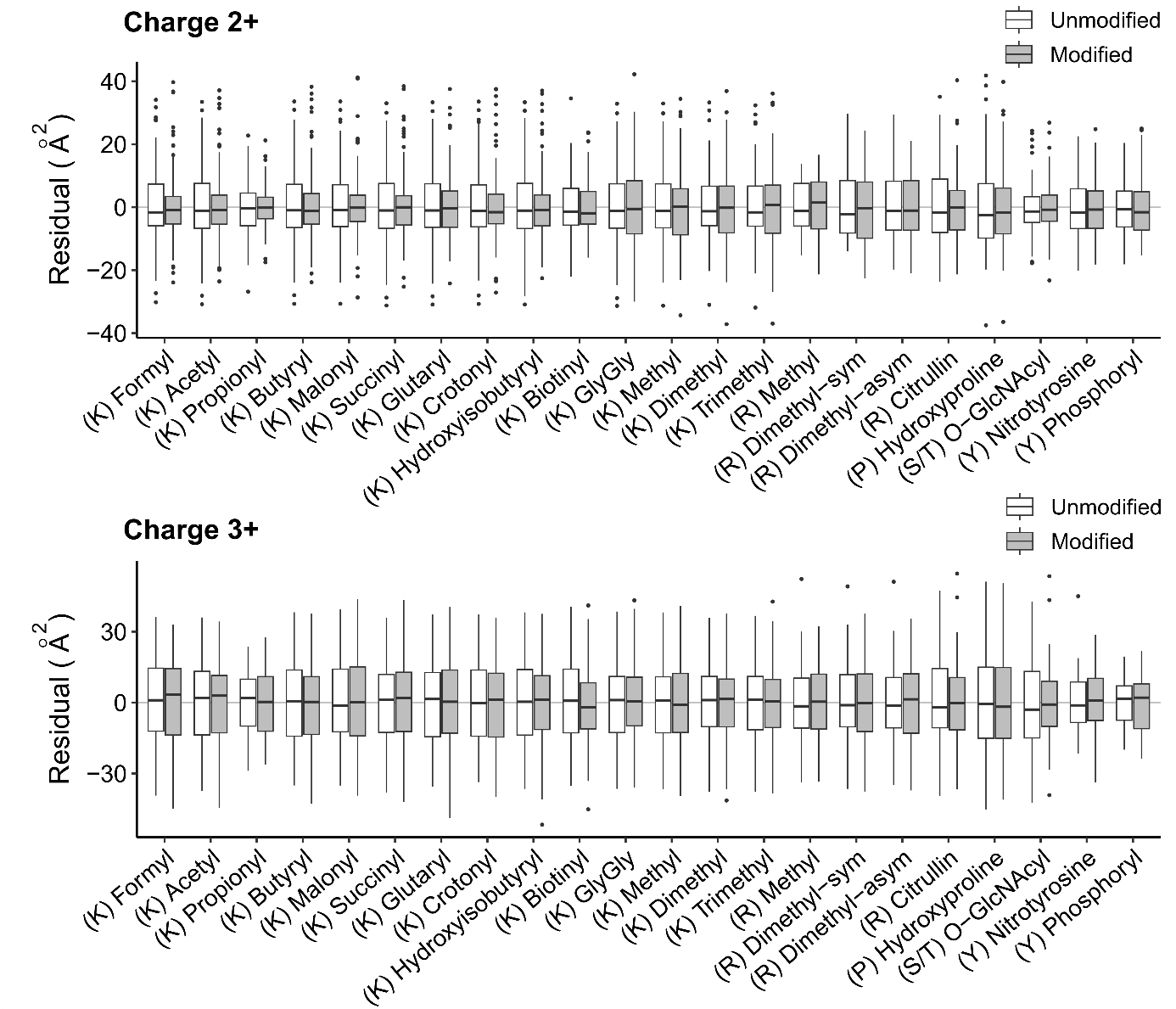


**Supplementary Figure 8.** Residuals of logarithmic regression for modified and unmodified matched peptide populations at charge 2 and 3. The number of matched peptides for each modification is shown in Figure 4a.

**Supplementary Table 1.** Details on experimental parameters used in this study. Layout according to Gabelica V, et al. Mass Spectrom Rev. 2019;38(3):291-320.

|  | **Parameter** | **Part of or influences the definition of the measurand (IS = ion structure, gas, T and/or E/N** | **Values** |
| --- | --- | --- | --- |
| 1 | Analyte | IS | Pooled synthetic peptides |
| 2 | Solvent of LC effluent | May influence IS | Buffer A: 100% water + 0.1% formic acid Buffer B: 100% ACN + 0.1% formic acid  All buffers at LC-MS grade |
| 3 | Ionization method | May influence IS | Nano electrospray (Bruker CaptiveSpray) |
| 4 | Ionization polarity | Influences IS | Positive |
| 5 | Adduct ID | Influences IS | [M+2H]2+, [M+3H]+3, [M+4H]4+, MaxQuant result tables (evidence.txt) |
| 6 | Pre-IM ion transfer conditions | May influence IS | Instrument: Bruker timsTOF Pro / Bruker timsTOF HT with dual TIMS cartridge. Full method details are included in each raw file. |
| 7 | Post-IM ion transfer conditions | No, but critical for peak assignment of analytes that may fragment after IM. | See Supplementary Table 2 for experimental parameters and the Methods section for search parameters. |
| 8 | Method of measurement | No | TIMS 1/K0 values were calibrated linearly using three ions from the Agilent ESI LC/MS tuning mix (m/z, 1/K0: 622.0289, 0.9848 Vs cm^-2^; 922.0097, 1.1895 Vs cm^-2^; 1221.9906, 1.3820 Vs cm^-2^)  CCS values were calculated from 1/K0 values using the Mason Schamp equation and assuming T = 305 K and N2 as collision partner (m = 28 Da) |
| 9 | IM gas nature (incl. purity) | gas | N2 (ambient air) |
| 10 | IM gas temperature | T and influences IS | Not controlled (T ~ 305 K) |
| 11 | IM gas pressure | Influences E/N | Pressure at tunnel inlet ~2.4 mbar |
| 12 | Electric field | Influences E/N | linear scan range: 1.51 Vs cm^-2^ to 0.6 Vs cm^-2^ Voltage difference: 146 V (timsTOF Pro), 165 V (timsTOF HT) |
| 13 | Length of drift tube | Influences E/N | Not applicable |
| 14 | E/N | E/N | Not determined |
| 15 | IM separation time | May influence IS | 100 ms |
| 16 | Calibrant or QC compounds | No | Low concentration Agilent ESI LC/MS tuning mix m/z, 1/K0: 622.0289, 0.9848 Vs cm^-2^; 922.0097, 1.1895 Vs cm^-2^; 1221.9906, 1.3820 Vs cm^-2^ |

**Supplementary Table 2.** Source and ion transfer parameters used in this study.

|  | **Parameter** | **Values** |
| --- | --- | --- |
| Source | End Plate offset | -500 V |
|  | Capillary | 1750 V |
|  | Nebulizer | 0 |
|  | Dry gas | 3 l/min |
|  | Dry Temp | 180 °C |
| Transfer | Deflektion1 Delta | 70.0 V |
|  | Funnel 1 RF (timsTOF Pro)  Funnel 1 RF | 500 Vpp |
|  | Funnel 2 RF | 200 Vpp |
|  | Multipole RF | 500 Vpp |
| TIMS parameter sets | 1/K0 | 0.6–1.5 Vs/cm^2^ |
|  | Ramp time | 100 ms |
|  | Accumulation time | 100 ms |
|  | Duty cycle | 100% |
|  | Cycle time | 1.17 ms |
|  | TIMS delta t1 | -20 V |
|  | TIMS delta t2 | -160 V |
|  | TIMS delta t3 | 90 V |
|  | TIMS delta t4 | 110 V |
|  | TIMS delta t5 | 0 |
|  | TIMS delta t6 | 55 V |
|  | Collision cell in (timsTOF HT)  Collision cell in (timsTOF Pro) | 220 V  220 V |

**Supplementary Table 3.** Parameters for MaxQuant modification search.

| **Modification** | **Mass (Da)** | **Residue** | **Composition** |
| --- | --- | --- | --- |
| Acetylation | 42.011 | K | H(2) C(2) O |
| Biotinylation | 226.078 | K | H(14) C(10) N(2) O(2) S |
| Butyrylation | 70.042 | K | H(6) C(4) O |
| Crotonylation | 68.026 | K | H(4) C(4) O |
| Dimethylation | 28.031 | K | H(4) C(2) |
| Formylation | 27.995 | K | C O |
| Glutarylation | 114.032 | K | H(6) C(5) O(3) |
| GlyGly | 114.043 | K | H(6) C(4) N(2) O(2) |
| Hydroxyisobutyrylation | 86.037 | K | H(6) C(4) O(2) |
| Malonylation | 86.000 | K | H(2) C(3) O(3) |
| Methylation | 14.016 | K | H(2) C |
| Propionylation | 56.026 | K | H(4) C(3) O |
| Succinylation | 100.016 | K | H(4) C(4) O(3) |
| Trimethylation | 42.047 | K | H(6) C(3) |
| Hydroxyproline | 15.990 | P | O |
| Citrullination | 0.984 | R | H(-1) N(-1) O |
| Dimethylation-asymmetric | 28.031 | R | H(4) C(2) |
| Dimethylation-symmetric | 28.031 | R | H(4) C(2) |
| Methylation | 14.016 | R | H(2) C |
| O-GlcNAcylation | 203.079 | S/T | H(13) C(8) N O(5) |
| Nitration | 44.985 | Y | H(-1) N O(2) |
| Phosphorylation | 79.966 | Y | H O(3) P |

**Supplementary Table 4.** Mean values and standard deviation (SD, n=3) for collision cross section (CCS) and retention time (RT) as well as the mass (m) of 11 iRT peptides. CCS was externally calibrated prior to each injection.

| **Sequence** | **1/K0 mean (V·s/cm^2^)** | **SD_1/K0_ (V·s/cm^2^)** | **CCS mean (Å^2^)** | **SD_CCS_ (Å^2^)** | **RT (min)** | **SD_RT_ (min)** | **m (Da)** |
| --- | --- | --- | --- | --- | --- | --- | --- |
| LGGNEQVTR | 0.79 | 4.16E-03 | 322.19 | 1.69 | 20.46 | 0.72 | 974.51 |
| YILAGVENSK | 0.87 | 6.26E-03 | 351.46 | 2.54 | 50.19 | 0.74 | 1094.60 |
| GTFIIDPGGVIR | 0.91 | 4.67E-03 | 370.27 | 1.89 | 85.49 | 0.34 | 1245.71 |
| GTFIIDPAAVIR | 0.92 | 4.12E-03 | 373.82 | 1.67 | 96.30 | 0.11 | 1273.74 |
| GAGSSEPVTGLDAK | 0.93 | 5.61E-03 | 375.23 | 2.27 | 38.46 | 0.76 | 1289.65 |
| TPVITGAPYEYR | 0.96 | 4.11E-03 | 387.71 | 1.66 | 59.92 | 0.50 | 1367.71 |
| TPVISGGPYEYR | 0.96 | 5.60E-03 | 388.16 | 2.27 | 55.81 | 0.57 | 1339.68 |
| ADVTPADFSEWSK | 0.97 | 5.59E-03 | 392.19 | 2.26 | 70.93 | 0.41 | 1453.67 |
| VEATFGVDESNAK | 0.98 | 2.69E-03 | 394.96 | 1.09 | 45.26 | 0.88 | 1367.66 |
| DGLDAASYYAPVR * | 0.99 | 5.58E-03 | 401.03 | 2.26 | 66.38 | 0.84 | 1398.68 |
| LFLQFGAQGSPFLK | 1.05 | 4.62E-03 | 423.71 | 1.87 | 99.82 | 0.06 | 1553.86 |

*In some LC-MS experiments, MaxQuant assigned two ‘evidences’ to this peptide sequence. In these cases, we selected the earlier eluting peak, which was in good agreement with the linear retention time model provided by the supplier of the iRT peptides.

**Supplementary Table 5**. Aligned mean CCS, standard deviation (SD), and coefficient of variation (CV) across 84 measurements of 11 iRT peptides.

| **Sequence** | **CCS mean (A^2^)** | **SD (A^2^)** | **CV (%)** |
| --- | --- | --- | --- |
| LGGNEQVTR | 321.78 | 1.75 | 0.54 |
| YILAGVENSK | 351.57 | 1.17 | 0.33 |
| GTFIIDPGGVIR | 369.22 | 0.86 | 0.23 |
| GTFIIDPAAVIR | 372.64 | 0.97 | 0.26 |
| GAGSSEPVTGLDAK | 374.92 | 1.41 | 0.38 |
| TPVISGGPYEYR | 389.24 | 1.17 | 0.30 |
| TPVITGAPYEYR | 389.81 | 1.35 | 0.35 |
| ADVTPADFSEWSK | 392.99 | 1.02 | 0.26 |
| VEATFGVDESNAK | 393.95 | 0.88 | 0.22 |
| DGLDAASYYAPVR | 401.65 | 1.35 | 0.34 |
| LFLQFGAQGSPFLK | 423.14 | 1.16 | 0.27 |
